# Feasibility of a circulation model for the assessment of endovascular recanalization procedures and periprocedural thromboembolism *in-vitro*

**DOI:** 10.1038/s41598-019-53607-2

**Published:** 2019-11-22

**Authors:** René Rusch, Jens Trentmann, Lars Hummitzsch, Melanie Rusch, Schekeb Aludin, Assad Haneya, Martin Albrecht, Jost Philipp Schäfer, Thomas Puehler, Jochen Cremer, Rouven Berndt

**Affiliations:** 1 Department of Cardiovascular Surgery, University Hospital of Schleswig-Holstein, Kiel, Germany; 2 Department of Radiology and Neuroradiology, University Hospital of Schleswig-Holstein, Kiel, Germany; 3 Department of Anesthesiology and Intensive Care Medicine, University Hospital of Schleswig-Holstein, Kiel, Germany; 4 Department of Orthopedics and Trauma Surgery, University Hospital of Schleswig-Holstein, Kiel, Germany

**Keywords:** Embolism, Experimental models of disease, Preclinical research

## Abstract

Aim of this study was to establish a simple and highly reproducible physiological circulation model to investigate endovascular device performance. The developed circulation model included a pneumatically driven pulsatile pump to generate a flow rate of 2.7 L/min at 70 beats per minute. Sections from the superficial femoral arteries were used in order to simulate device/tissue interaction and a filter was integrated to analyze periinterventional thromboembolism of white, red and mixed thrombi. The working fluid (3 L) was a crystalloid solution constantly tempered at 36.5 °C. To evaluate the model, aspiration thrombectomy, stent-implantation and thrombectomy with the Fogarty catheter were performed. Usability of the model was measured by the System Usability Scale (SUS) – Score. Histological specimens were prepared and analyzed postinterventional to quantify tissue/device interaction. Moreover, micro- and macroembolism were evaluated for each thrombus entity and each device. Results were tested for normality using the D’Agostino-Pearson test. Statistical comparisons of two groups were performed using the Student’s t-test. All devices were able to remove the occlusions after a maximum of 2 attempts. First-pass-recanalization was not fully achieved for aspiration thrombectomy of mixed thrombi (90.6%), aspiration thrombectomy of red thrombi (84.4%) and stent-implantation in occlusions of red thrombi (92.2%). Most micro- and macroembolism were observed using the Fogarty catheter and after stent-implantation in occlusions of white thrombi. Histological examinations revealed a significant reduction of the vascular layers suggesting vascular damage after use of the Fogarty catheter (327.3 ± 3.5 μm vs. 440.6 ± 3.9 μm; p = 0.026). Analysis of SUS rendered a mean SUS-Score of 80.4 which corresponds to an excellent user acceptability of the model. In conclusion, we describe a stable, easy to handle and reproducible physiological circulation model for the simulation of endovascular thrombectomy including device performance and thromboembolism.

## Introduction

During the last decade, endovascular recanalization of thrombotic occlusions has achieved greater success through improvement in device technology and the technical abilities of interventionalists^1^. The extended use of mechanical percutaneous thrombectomy devices like Rotarex® and Angiojet® and the widespread use of various covered and uncovered stent systems marks a new era of interventional percutaneous thrombectomy and expands the scope of action for both arterial and venous disease and both vascular surgeons and vascular interventionalists far beyond the classical surgical thrombectomy^2,3^. Therefore, all vascular disciplines have made great efforts to establish suitable and standardized circulation models for endovascular training, device testing and experimental studies^4,5^. But until today, none of these models fulfil the special requirements of simulating percutaneous thrombectomy including thromboembolism, vascular endothelium and different types of thrombi. Most of these described circulation models include plastic replica of the vascular tree and only a few models provide cadaveric vessels with or without a crystalloid solution pumped through the model^4,5^. Although these models allow the training of basic endovascular procedures, they do not simulate satisfactorily the human vascular system and usually do not allow the sufficient experimental testing of new endovascular devices and new imaging procedures. Moreover, most of these models have never been evaluated or standardized.

Further, a number of issues like postinterventional thromboembolism, vascular endothelium/device interaction, different thrombus entities and large-scale experiments of recanalization could not be investigated sufficiently in groups of large animals or human corpses.

Nevertheless, a standardized and fully evaluated *in-vitro* circulation model for simulating thrombectomy of thrombotic occlusions/stenosis with different thrombus entities and potential thromboembolism has not been established so far. Ideally, the proposed circulation model should be adapted to the following issues: (I) the model should represent sufficient and reproducible physiological flow conditions with a blood substitute, (II) the model should enable good technical manageability for the interventionalist simulating different segments of the human arterial tree with multilayered vessels with potential for dissection and damage of the vascular endothelium, (III) the model should include different entities of thrombi and periinterventional thromboembolism.

## Material and Methods

### Development of the circulation model

**Figure 1:**
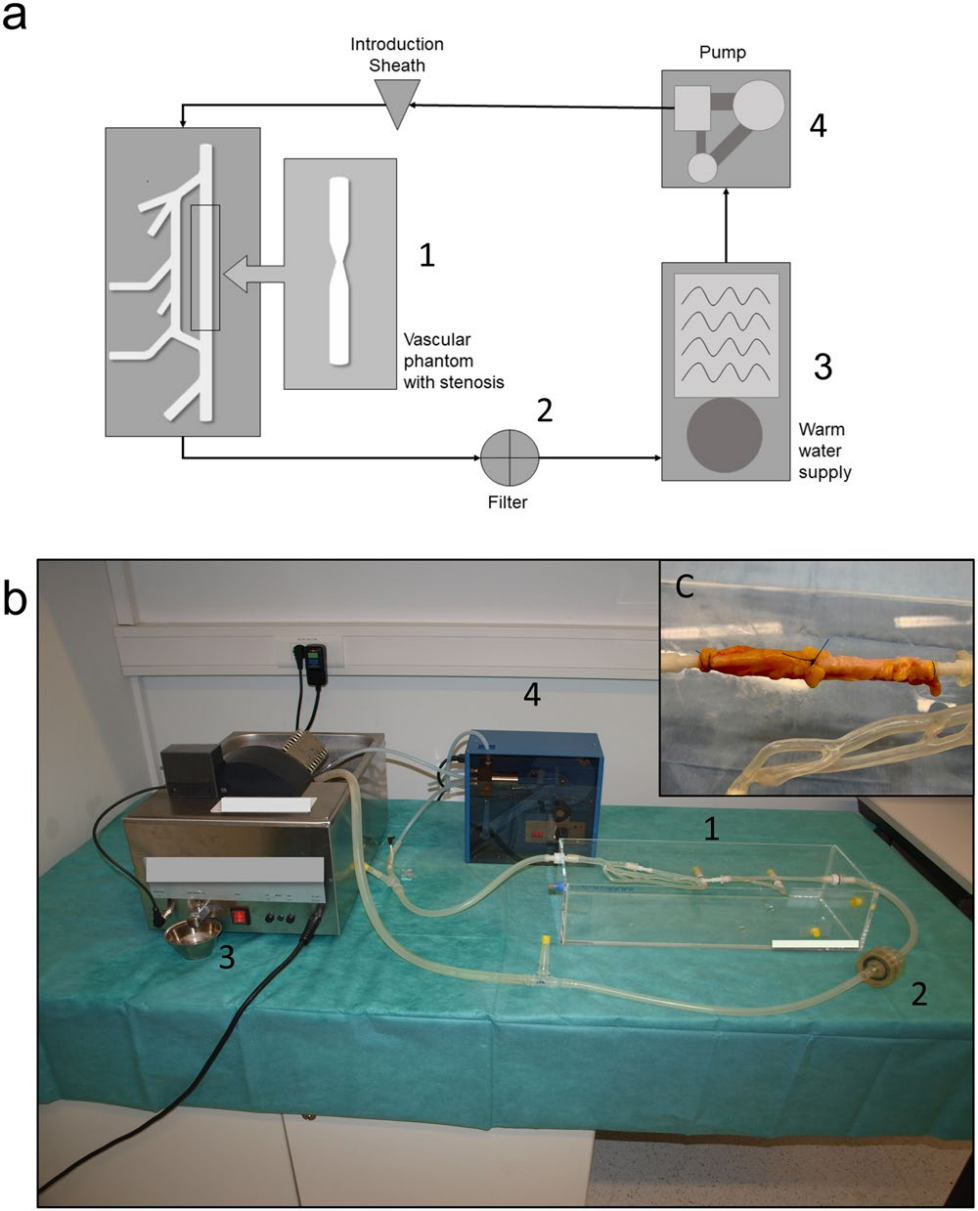
Schematic representation (**a**) and downsized visualization (**b**) of the circulation model: (1) replaceable section for the (**c**) vascular-phantom or the whole arterial tree, (2) filter for collecting thromboembolism, (3) warm water supply, (4) pneumatically driven pulsatile pump. The working fluid (3 L) consisted of a crystalloid solution (60% glucose solution). Depiction of the vacular phantom used for the evaluation of device/tissue interaction.

To achieve a physiological circulation model of the human arterial tree, a mechanical volume-controlled pulsatile pump (modified 7025 Rodent Ventilator; Ugo Basile, Gemonio, Italy) was used to generate a flow rate of 2.7 L/min at 70 beats per minute and with a peak flow at 3.3 L/min (Fig. 1a,b). Sections from the human superficial femoral artery (SFA) were provided by voluntary donors undergoing transfemoral amputation and were then transferred into an acrylic resin-based model of the femoropopliteal section (ELASTRAT, Geneva, Switzerland).

After extraction all vessels were examined sonographically within the circulation model. High grade stenosis (>50%) as well as occluded vessels and/or plaque ruptures were removed from the study. The extracted vessels had a standard length of 60 mm and a vessel diameter of 6.38 ± 3.17 mm. To fix and stabilize the thrombus position within the vascular phantom, a simple ligature of the extracted vessel was performed (standardized via Hegar probe) in central position of the extracted vessel (Fig. 1c). The Hegar probe defined a standardized residual lumen (5 mm) of the vascular phantom in order to guarantee both a stable thrombus position and an unhindered passage of the endovascular devices. Thrombus position and residual vascular lumen was verified sonographically under flow conditions.

The working fluid (3 L) consisted of a crystalloid solution (60% glucose solution). Alternatively, in former experiments we have also used a suspension of water and whole blood at a volume ratio of 1:3 in order to simulate long-term lysis with pharmacological agents and imaging procedures for intraluminal navigation^6^. The working fluid was constantly tempered at 36.5 °C by warm water supply. Temperature and intraluminal pressure of the model were constantly measured via an arterial pressure (D19 KT Display; GE Medical Systems, Chicago, USA and DPT-6000 pressure-set; CODAN Critical care GmbH, Forstinning, Germany) and temperature probe (Testo 108; Testo SE and Co KGaA, Lenzkirch, Germany). Periinterventional thromboembolism was determined by collecting micro- and macroembolism in a filter from an extracorporeal circulation system (Schleicher und Schüll, Düren, Germany).

### Experimental protocol

This feasibility study serves to develop and evaluate an *in-vitro* circulation model for studying endovascular thrombectomy including device/tissue interaction and periinterventional thromboembolism (Fig. 1a,b). Therefore, four endovascular trained surgeons evaluated the model and performed aspiration thrombectomy (n = 8/each investigator), thrombectomy with a Fogarty catheter (n = 8/each investigator) and stent-implantation (n = 8/each investigator). All experiments were performed in a hybrid-operation room. Usability of the model was measured by the System Usability Scale (SUS) - Score, as previously described^7^.

To evaluate potential dissection and/or damage of the vascular endothelium, histological samples were taken and the number of micro- and macroembolisms were analyzed. To evaluate the basic functionality of the model, intraluminal pressure and temperature measurement was continuously performed via an arterial pressure system (D19 KT Display; GE Medical Systems, Chicago, USA and DPT-6000 pressure-set; CODAN Critical care GmbH, Forstinning, Germany) and a temperature probe (Testo 108; Testo SE and Co KGaA, Lenzkirch, Germany). The Studies have been approved by the local ethical committee of the University Medical Center Schleswig-Holstein, Kiel, Germany (protocol identification: D513/19). This research was in compliance with the Helsinki Declaration and informed written consent was obtained from all donors/participants.

### Generation of different thrombus entities and preparation of the intraluminal thrombus

As previously reported, thrombi were made in the Chandler loop under standardized conditions^8,9^. Venous blood was taken from five voluntary, healthy male donors and haematologic testing was performed prior to blood sampling including testing for Von Willebrand disease, haemophilia, the factor V Leiden mutation and thrombophilia diagnosis. A polymeric vessel-phantom was used to obtain standardized thrombus dimensions (length = 30 mm, diameter = 6 mm) from the Chandler loop. A series of former experiments and pretests (n = 150) was performed to secure an adequate reproducibility of the thrombi^6^. The thrombi were extracted, measured and immediately transferred into the circulation model.

Red thrombi (agglutinative thrombi) were generated by using human full blood, white thrombi (precipitation thrombi) were generated by using human plasma and mixed thrombi (white and red) were generated by using human full blood and plasma at a volume ratio of 1:1^10^.

### Endovascular procedures

Four endovascular trained surgeons evaluated the model and performed the endovascular procedures in the circulation model. An introduction sheet was inserted proximal and the thrombotic occlusion was passed via a 0.035 inch guide wire (Terumo GmbH Germany, Eschborn, Germany) in order to perform simple aspiration thrombectomy, stent-implantation (Abbott, Chicago, USA; Absolut Pro Vascular self-expanding stent, 6 × 40 mm) and thrombectomy via Fogarty catheter (Edwards Lifesciences, Irvine, USA; Over-the-wire Fogarty catheter). Afterwards all interventionalists answered the SUS questionnaire and evaluated the handling of the model.

### Analysis of thromboembolism

Micro- and macroembolism were collected in a post-occlusion filter (Schleicher und Schüll, Düren, Germany) distal of the simulated thrombus. After each recanalization maneuver, the post-occlusion filter was checked for embolized thrombus fragments by a blinded observer via microscope. Fragments > = 1 mm (macroemboli) and <1 mm (microemboli) were separately counted.

### Histological analysis

Six proximal, mean and distal vessel specimens were routinely fixed in 4% formalin solution and embedded in paraffin. Paraffin-embedded vascular tissue sections (4 μm) were then stained with hematoxylin-eosin (HE) to define the intimal and medial layers by light microscopy. The intima-media-thickness was quantified in ten random vascular sections by an image analyzer (Samba 2000^®^; North Sioux City, Iowa, USA).

### Statistical analysis

All values are expressed as mean ± standard deviation (SD). Categorical variables are presented as frequency distributions (n) and percentages (%). Data were analyzed with Graph Pad Prism version 5.01 for Windows (GraphPad Software; San Diego, California, USA) and tested for normality using the D’Agostino-Pearson test. Statistical comparisons of two groups were performed using the Student’s t-test. A p-value < 0.05 was considered as significant.

## Results

### Performance of endovasculare procedures and usability of the circulation model

**Figure 2:**
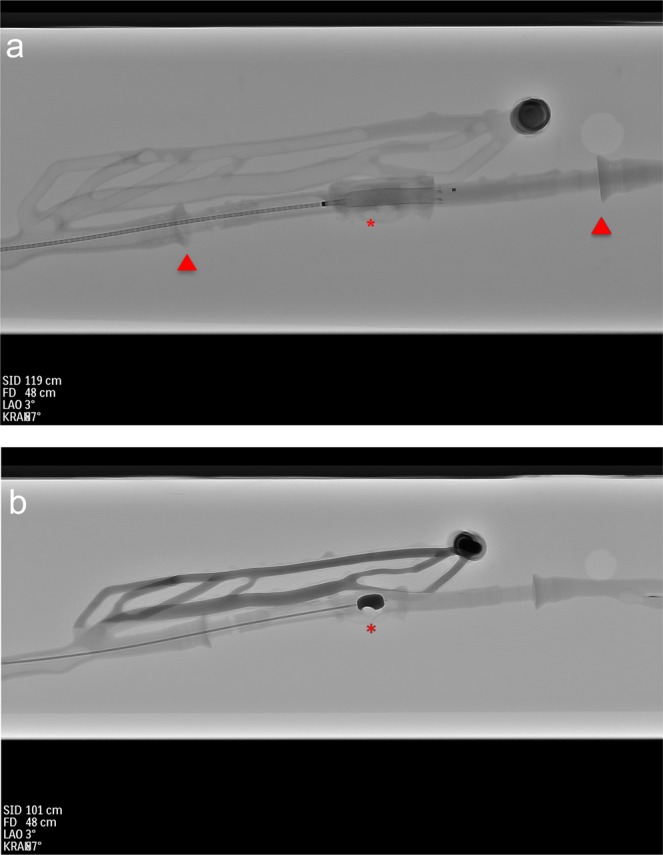
Representative imaging of thrombectomy performed in the circulation model. Performance of stent-implantation (**a**) in the region of the thrombotic occlusion. Performance of thrombectomy with the Fogarty catheter (**b**). Red arrows mark the position of the replaceable vascular section. Red asterisks show the position of the stent respectively the Fogarty catheter.

Endovascular recanalization was achieved in 92 out of the 96 maneuvers. Four maneuvers failed because of mishandling of the model (n = 2) and the device (n = 2). All devices (aspiration catheter, Fogarty catheter, self-expanding stent) were able to remove or eliminate (stent implantation) the thrombus after a maximum of 2 attempts (Fig. 2). First-pass-recanalization was not fully achieved for aspiration thrombectomy of mixed clots (90.6%), aspiration thrombectomy of red thrombi (84.4%) and stent-implantation in occlusion of red thrombi (92.2%), as presented in Tables 1–3. The evaluation of the circulation model rendered a mean SUS-Score of 80.4 which corresponds to a percentile rank of 90% and therefore an excellent acceptability range/adjective rating^7^. Mean intraluminal pressure was 83.5 mm/Hg ± 4.32 and temperature was 36.43 C ± 2.23 measured at six different time points during the experiments.

**Table 1:** Endovascular removal or elimination of white thrombi.

	First-pass-recanalization (%)	Macroemboli (n)	Microemboli (n)
aspiration catheter	100	0	1
Fogarty catheter	100	8	11
Stent implantation	100	6	9

**Table 2:** Endovascular removal or elimination of red thrombi.

	First-pass-recanalization (%)	Macroemboli (n)	Microemboli (n)
aspiration catheter	84.4	1	2
Fogarty catheter	100	5	8
Stent implantation	92.2	3	6

**Table 3:** Endovascular removal or elimination of mixed thrombi.

	First-pass-recanalization (%)	Macroemboli (n)	Microemboli (n)
aspiration catheter	90.6	2	4
Fogarty catheter	100	3	6
Stent implantation	100	4	6

### Analysis of thromboembolism

**Figure 3:**
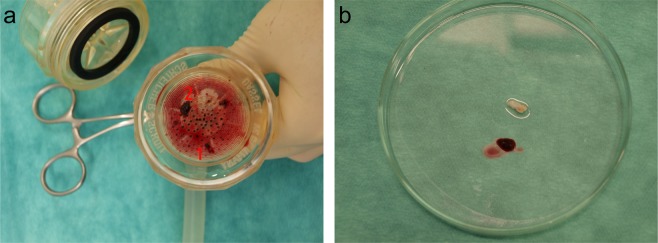
Postinterventional analysis of micro (1) - and macroembolism (2) after opening the post-occlusion filter (**a**) and representative imaging of red and white thrombi after thrombectomy with the Fogarty catheter (**b**).

Micro- and macroembolism occur in all endovascular maneuvers except aspiration thrombectomy of red thrombi (Tables 1–3). Most micro- and macroembolism were observed using the Fogarty catheter (n = 11) and stent-implantation (n = 9) in occlusions of white thrombi (Tables 1–3; Fig. 3).

### Histological analysis

Specimens of arteries were stained with HE to examine potential dissection and damage of the vascular layers. No macroscopic dissection was observed in the current experiments. The intima-media-thickness did not differ significantly after aspiration, thrombectomy and stent-implantation. However, histological examination revealed significant vascular lesions of tunica intima and media after using the Fogarty catheter (327.3 ± 3.5 μm vs. 440.6 ± 3.9 μm; p = 0.026; Supplement 1).

## Discussion

Endovascular procedures have marked a new era of vascular medicine during the last two decades^11,12^. Accordingly, endovascular methods have also been performed to recanalize thrombotic occlusions by mechanic thrombectomy or stent-implantation but until today no suitable *in-vitro* model for the simulation of thrombotic occlusions and thromboembolism is available^4,5^. Most of the so far described *in-vitro* models allow the basic training of endovascular procedures and simple experimental testing but they do not simulate satisfactorily the human vascular tree and circulation system. Therefore, these models usually do not allow the sufficient experimental testing of new endovascular devices respectively new imaging procedures including catheter handling, thromboembolism and injury of the vascular layers.

In contrast, animal models can offer a physiological circulation, however the size of most animal vessels is too small for many devices being introduced to human arteries. Larger mammals like pigs and sheep could simulate the human arterial tree, but experiments with these animals are subjected to major logistic challenges, ethical concerns and great expense^4,13^. Moreover, a number of issues like postinterventional thromboembolism, vascular endothelia/device interaction, different thrombus entities and large-scale experiments of vascular recanalization cannot be investigated sufficiently in groups of large animals.

The reproducible model featured in this study provides a physiological circulation, easy access for the interventionalist, simulation of vascular-tissue/device interactions and analysis of periinterventional thromboembolism due to different thrombus entities. Accordingly, the analysis of the SUS-score originally created to provide a simple assessment of technical procedures, revealed a high acceptability range and support our hypothesis of high user acceptance^7^. All participating investigators attested an excellent technical performance and acceptability range of the circulation model. Therefore, the model should be easily reproducible and usable for other researches in the vascular field.

The basic functionality (temperature, blood pressure) of the circulation system was assessed at six time points during the evaluation experiments and the reproducibility and stability of the model was particularly high (SD <10%). The pilot experiments showed that complete first-pass-recanalization was achieved for all devices except aspiration thrombectomy of mixed and red thrombi which corresponds to the consistence of the different thrombus entities. Agglutinative “red” thrombi are composed of cellular deposits and feature a more rigid surface whereas “white” precipitation thrombi grow out of the plasmatic components with a softer structure. Consequently, the pilot experiments showed that micro- and macroembolism differed between the three methods of thrombectomy and also between the three types of thrombi reflecting that the choice of the vascular tool and composition of the thrombus impact significantly thromboembolism.

One major objective of the current study was to establish a model which enables the investigation of tissue/device interaction under physiological conditions. Histological specimens were prepared after the experiments and revealed that only the use of the Fogarty catheter was associated with significant damage of the vascular layer which is supported by the findings of former studies for mechanical thrombectomy^14^.

Therefore, the model also provides differentiated results of device performance which seems necessary as development in vascular imaging provides, in the future, more precise assessment of thrombus localization and composition and market offers the clinician a bewildering number of vascular tools^15,16^.

In conclusion the here developed circulation model which can be easily established and employed by other groups offers three major benefits: (I) a stable, easy to handle and reproducible physiological circulation for the simulation of endovascular access (II) a cadaveric vascular tree/vascular interposition for studying the influence of endovascular tools on the vascular layer; and (III) the possibility to simulate agglutinative thrombotic occlusions or precipitation thrombi and easy assessment of perioperative thromboembolism.

## Supplementary information

Supplement 1
