# Supplementary material for: Feasibility of a circulation model for the assessment of endovascular recanalization procedures and periprocedural thromboembolism in-vitro
Source: Sci Rep. 2019 Nov 22;9:17356. doi: 10.1038/s41598-019-53607-2 (PMC6874641; doi:10.1038/s41598-019-53607-2)
Supplement: Supplementary file 1 — Supplement 1 [file 41598_2019_53607_MOESM1_ESM.pdf]

Feasibility of a circulation model for the assessment of endovascular recanalization procedures and periprocedural thromboembolism *in-vitro*

René Rusch[1]*, Jens Trentmann[2]*, Lars Hummitzsch[3], Melanie Rusch[4], Schekeb Aludin[2], Assad Haneya[1], Martin Albrecht[3], Jost Philipp Schäfer[2], Thomas Puehler[1], Jochen Cremer[1], Rouven Berndt[1]*

[1] Department of Cardiovascular Surgery, University Hospital of Schleswig-Holstein, Kiel, Germany

[2] Department of Radiology and Neuroradiology, University Hospital of Schleswig-Holstein, Kiel, Germany

[3] Department of Anesthesiology and Intensive Care Medicine, University Hospital of Schleswig-Holstein, Kiel, Germany

[4] Department of Orthopedics and Trauma Surgery, University Hospital of Schleswig-Holstein, Kiel, Germany

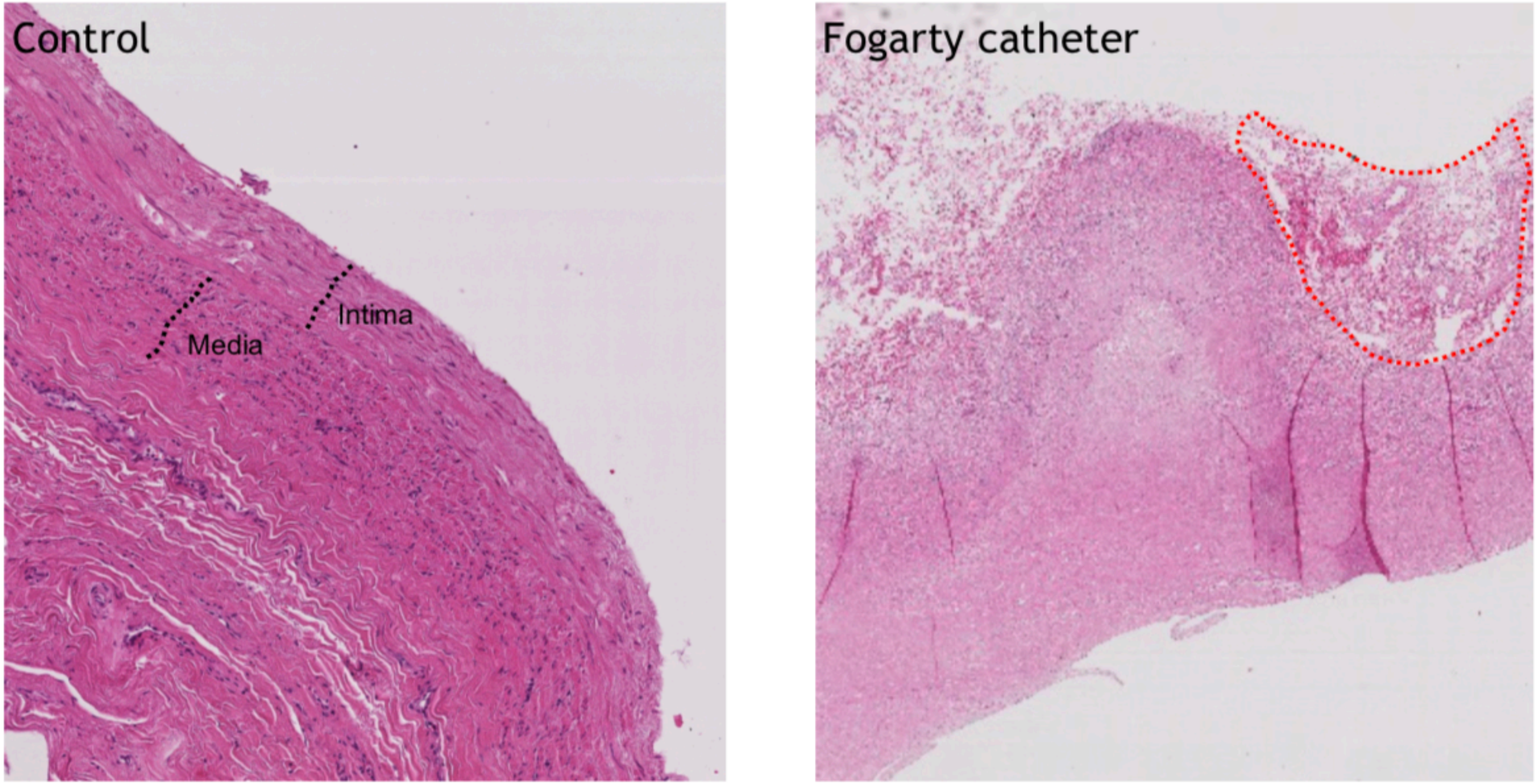

Supplement 1

Supplement 1

Representative imaging of the histological analysis. Black lines mark the intima and media (control). Red lines define the intima/media lesions after thrombectomy with the Fogarty catheter (original magnification 200x).
